# 

**Published:** 1916-02-05

**Authors:** 


### CONTENTS.

PAGE

**The Will to Recover** ... ... ... ... ... 399

Its Victories and Limitations.

**The Numbers of Practitioners and Students**... 400

**Hospital and Institutional News** ... ... ... 401
Will Every Reader Please Help?—A Loss to Charing Cross Hospital—A Secretary's Salary Revised—Some Absurdities of Ignorance—An Institutional Personality—Miss Eva Lückes—Anti-Enteric Inoculation—A Medical Inspector's Salary—Hackney Red Cross Hospital—A Record Distribution—The Central Midwives Board's Arbitrariness—An Indictment of the Bradford Clergy—The Finance of the Insurance Act—An Institutional Will—Medical Men and the Compulsion Bill—The Right to Postpone the Realisation of Securities—Death of Two Medical Knights—More Frightfulness?—Medical Literature and the War—The Royal Free Hospital Unit in Serbia—A Dispensary Secretary's Long Record—The London General Hospitals: A Reduction of *à la suite* Staffs—Cotton Waste Replaced by Rags—Mental Hospital Attendants and War Service—"Kitchener's Navy"—Empire Hospital Country Home—St. Katharine's and the East End—The Employment of Consumptives—This Week's Drug Market.

**The Medical Examination of Recruits** ... ... 407
An Effort to Amend the Muddle.

**Tobacco and the Soldier** ... ... ... ... 408

The Medico-Military Problem.

**Hospitals and the Poets** ... ... ... ... 409

III.—Keats' Seven Years of Medicine.

**Round the World** ... ... ... ... ... ... 410

**The Institutional Library** ... ... ... ... 411
The Rise of Rail-Power in War and Conquest, 1833 to 1914—English Public Health Administration—Medical Emergencies—Instinct and Intelligence—The Seven Ages of Woman—The Structure of the Fowl—The Wounded French Soldier.

**The Department of Nursing** ... ... ... 413
Matrons and Sisters.
Round the Hospitals.

**Textile Supplies for Hospitals** ... ... ... 415
IV.—The Animal Fibres.

**The Heating of Hospitals** ... ... ... 417
VII.—Bristol Royal Infirmary.

**Parliament and the Profession** ... ... ... 419

**Passing Events and Topics** ... ... ... ... 419

**The Editor's Letter-Box** ... ... ... ... 420

**The Institutional Worker** ... ... (Suppl.) 1
Gas Irons in Laundries.
Food Economy and Its Difficulties.
Questions for February: (1) A Hospital or Institution Invention—(2) Economies in the Matron's Department.

